# Dynamic Changes and Comprehensive Evaluation of Agronomic Traits and Nutritional Quality of *Cichorium intybus* at Different Growth Stages

**DOI:** 10.3390/plants15050837

**Published:** 2026-03-09

**Authors:** Xiaolu Ma, Yunxia Ma, Guang Yang, Yazhou Shao, Gangtie Li, Xiandong Meng, Shuai Zhang

**Affiliations:** 1 College of Desert Control Science and Engineering, Inner Mongolia Agricultural University, Hohhot 010019, China; 2 State Key Laboratory of Water Engineering Ecology and Environment in Arid Area, Inner Mongolia Agricultural University, Hohhot 010019, China

**Keywords:** *Cichorium intybus*, growth stage, agronomic traits, nutritional quality, the best harvest time

## Abstract

Under the background of a supply gap expansion for high-quality forage grass in China and the high degree of dependence on foreign countries, it is necessary to clarify the best feeding and harvesting period for *Cichorium intybus* in the temperate continental monsoon climate zone of Northern China. To achieve this goal, this study systematically explored the agronomic traits and nutritional quality of *Cichorium intybus* during the nutritional period (June–July), flowering period (July–August), and fruiting period (August–September) in the Hohhot experimental base. We measured agronomic indexes, such as the plant height and basal stem, and nutrients, such as the dry matter (DM) and crude protein (CP), and calculated the total digestible nutrients (TDN) and other feeding value indexes. The results showed that the plant height of *Cichorium intybus* increased from 54.60 cm in the vegetative stage to 204.10 cm in the fruiting stage, and the fresh grass yield increased from 8775.045 kg/hm^2^ in the vegetative stage to 19,035.09 kg/hm^2^ in the fruiting stage. The DM content of the stems and leaves was the lowest (stem: 8.73%; leaf: 14.04%), but the CP (leaf: 20.32%) and crude fat (EE, leaf: 5.02%) contents were the highest. The TDN was 66.78%, the relative feed value (RFV) was 255.61, the comprehensive membership function value was 0.54 for the stems and 0.60 for the leaves, and the feeding value was the best. WSC accumulation was significant during the flowering stage; the fiber content of the DM (stem: 20.52%; leaf: 20.31%) and the acid detergent fiber (ADF, stem: 42.43%) were the highest during this stage; and the CP decreased to 10.97%. A correlation analysis showed that the plant height and stem diameter were significantly positively correlated with the yield and fiber accumulation. This study confirmed that the nutritional period was the best harvest period for obtaining high-protein and high-digestibility forage, and the fruiting period was suitable for processing hay or silage. These results provide a scientific basis for the large-scale feed development of *Cichorium intybus*.

## 1. Introduction

The steady development of animal husbandry is the key pillar for ensuring an adequate supply of meat, eggs, and milk in China and building a strong agricultural industrial system. As the core material basis of animal husbandry, the supply capacity of high-quality forage grass directly restricts the process of industrial upgrading [1]. At present, the market gap of high-yield and high-quality forage varieties in China is continuing to expand. The local supply is struggling to match the development needs of animal husbandry, and the dependence on foreign imports remains high [2]. Therefore, there is an urgent need to explore and develop local high-quality forage resources and optimize the forage supply structure to promote the sustainable development of the grass and livestock industries. *Cichorium intybus* is a perennial herb that is native to the Mediterranean, Central Asia, and North Africa [3,4]. It is a multi-purpose economic plant with edible, feeding, medicinal, and industrial application value [5,6,7]. In the field of feeding, *Cichorium intybus* shows outstanding advantages: its nutrient richness is close to or even greater than that of some alfalfa varieties, with a high yield, good palatability, a high digestibility, strong stress resistance, drought tolerance, and excellent disease and insect resistance [8,9,10]. In addition, feeding *Cichorium intybus* can reduce the probability of parasitic infections in livestock and significantly improve their production performance. Studies have shown that the production performance of livestock fed with *Cichorium intybus* is comparable to that of livestock fed with legumes, and is superior to a grass forage feeding group [11,12]. For example, the milk yield of cows fed with *Cichorium intybus* can be significantly improved, and the negative effects of repellents on livestock growth can be reduced [13,14].

The agronomic traits and nutritional quality of forage grass are regulated by multiple factors, such as the geographical location, climatic conditions, and field management. As a key link of human intervention, the harvest period can influence the synergistic optimization of yield and nutritional quality by adapting the phenological characteristics of forage grass and regional climate laws, thus providing sufficient and high-quality forage grass resources for livestock [15]. At present, the international research on *Cichorium intybus* has mostly focused on the influencing factors of yield and quality improvement. In 1990, Nicola et al. [16] explored the effects of different harvest periods on the yield and quality of two *Cichorium intybus* varieties in Italy. In 1999–2000, Rodkiewicz [17] conducted a study on the effect of the sowing date on the yield of *Cichorium intybus* in Poland. In China, *Cichorium intybus* has been introduced and cultivated in northern cold regions such as the Shaanxi–Gansu–Ningxia irrigation area, and has been widely planted in Guizhou, Sichuan, and other places [18,19,20]. Its good adaptability, low soil requirements, and characteristics that favor its intercropping with food crops provide an effective way to alleviate the contradiction between planting forage and food crops.

Although the feeding value of *Cichorium intybus* has been preliminarily verified, there are still obvious scientific gaps in the existing research. Firstly, there is a lack of systematic comparative data on the morphological characteristics, fresh grass yield, nutritional value, and feeding potential of *Cichorium intybus* during the three key growth stages—the vegetative stage, flowering stage, and fruiting stage—in northern temperate continental monsoon climate zones (such as Hohhot), and the core utilization value of *Cichorium intybus* during different growth stages is not clear. Secondly, the synergistic/restrictive relationship between the agronomic traits in different growth stages of *Cichorium intybus* and the internal relationship between agronomic traits and nutritional quality have not been deeply analyzed, which makes it difficult to guide targeted field management and harvest regulation. Thirdly, there is a lack of a comprehensive index-based *Cichorium intybus* optimal harvest period determination system; thus, there is no accurate scientific basis for achieving a balance between a “high quality” and a “high yield” in production practice.

Based on this, this study proposes the following scientific hypotheses: There are significant differences in the agronomic traits and nutritional quality of *Cichorium intybus* grass at different growth stages. The *Cichorium intybus* grass in the nutritional period has a higher level of core nutrients such as crude protein and crude fat, and the feeding value is optimal; as the growth period progresses, the fiber content will gradually increase and dry matter accumulation will increase, but the proportion of protein nutrients will decrease. The core objectives of this study were as follows: (1) to clarify the specific differences in the morphological characteristics, fresh grass yield, and nutritional value of *Cichorium intybus* grass during the three key stages of the vegetative period, flowering period, and fruiting period; (2) to determine the correlation between agronomic traits and the nutritional quality of *Cichorium intybus* at different growth stages; and (3) to identify the most suitable feed harvesting period for *Cichorium intybus* using a comprehensive evaluation. The results of this study will fill gaps in the research on the feeding value of *Cichorium intybus* at different growth stages for the specific climate zone of the north. This study also provides scientific support for the large-scale feeding development of *Cichorium intybus* grass and the efficient utilization of forage resources, and it helps with the optimization and upgrading of the animal husbandry forage supply system.

## 2. Materials and Methods

### 2.1. Research Area Overview

The experimental site was located at the experimental base of the College of Desert Control Science and Engineering, Inner Mongolia Agricultural University in Hohhot, with geographic coordinates of 110°46′–112°10′ E and 40°51′–41°8′ N. It is characterized by a mid-temperate, continental monsoon climate, with markedly distinct seasonal variations. Specifically, the region experiences long, harsh winters, short and hot summers, and rapid climate changes during the spring and autumn seasons. The average annual sunshine duration is 2840 h, distributed as 812 h in spring, 776 h in summer, 656 h in autumn, and 596 h in winter. The mean annual precipitation ranges from 350.5 to 427.5 mm, with an average of 392.2 mm across the city.

### 2.2. Experimental Design

#### 2.2.1. Plot Setup

**Figure 1:**
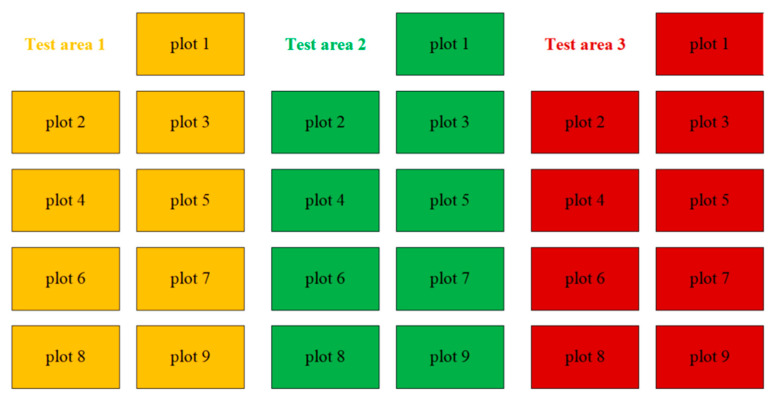
Distribution map of test area.

As shown in Figure 1, a randomized complete block design was used in the experiment, which was divided into three independent blocks (repeated) with an interval of 1 m between them. In each plot, nine sub-plots were established (one growth stage corresponded to three sampling sub-plots). Each sub-plot measured 5 m × 4 m = 20 m^2^, and the spacing between sub-plots was 0.5 m. The planting density of each plot was consistent with the original design (row spacing: 60 cm; plant spacing: 40 cm). The location of each sub-region was determined using a random number table to ensure random allocation. The total experimental area of the three sites was calculated as follows: 3 sites × 9 sub-blocks × 20 m^2^ = 570 m^2^.

#### 2.2.2. Sowing and Field Management

Sowing was carried out on 1 May 2023, with a sowing depth of 3–5 cm, a row spacing of 60 cm, and an inter-plant spacing of 40 cm. An irrigation plan was developed in accordance with local climate conditions and the growth characteristics of *Cichorium intybus*. Drip irrigation was implemented throughout the growing period. From sowing to the vegetative stage, irrigation was administered 2–3 times per week, with each irrigation event applying 20–30 L/m^2^. This approach ensured that the soil maintained adequate moisture to meet the water requirements of *Cichorium intybus* while avoiding root diseases caused by excessive water accumulation. The soil moisture was closely monitored during each irrigation event, and the water volume was adjusted based on the actual conditions. For fertilization management, a combination of compound fertilizer (N–P–K ratio of 15–15–15) and organic fertilizer (well-rotted cattle manure with an organic matter content ≥ 45%) was used. Prior to sowing, during soil preparation, the organic fertilizer was evenly broadcast over the experimental area at a rate of 3000 kg/hm^2^ and then incorporated into the soil, thereby providing long-lasting nutrient support and creating a favorable soil environment for *Cichorium intybus* growth.

#### 2.2.3. Delineation of Growth Stages and Sampling Periods

Based on phenological observations of *Cichorium intybus*, the original month-long growth period was refined into precise sampling intervals (each lasting 10–15 days), as follows:

(1) Trophophase: Plants attain a height of 30–40 cm with no bud formation; the sampling period was from 20 June to 30 June.
(2) Flowering period: More than 50% of the plants exhibit visibly detectable buds (diameter of 2–3 mm); the sampling period was from 20 July to 30 July.
(3) Bearing phase: The fruit has a hard outer shell with a stable color (e.g., deep brown, gray-black, etc.) and plump seeds with a significantly reduced moisture content. At this stage, gently rubbing the fruit causes the seeds to easily detach. The sampling period was from 20 August to 30 August.

### 2.3. Determination Methods

#### 2.3.1. Determination of Morphological Indicators

At each growth stage, 5 plants were randomly selected from each plot (a total of 15 plants) to measure indicators such as the plant height, basal stem, leaf count, tiller number, leaf area, and leaf relative water content.

(1) Plant height: measured using a tape measure from the base of the *Cichorium intybus* to the apex.
(2) Basal stem: measured with a vernier caliper at the second internode from the base of the stem.
(3) Leaf area: determined using an LI-3000A leaf area meter (LI-COR, Inc., Lincoln, NE, USA).
(4) Leaf relative water content: Following the method described by Gao Junfeng [21], one complete leaf with uniform vigor was selected from each plant. The fresh weight (Wf) of the leaf was recorded, and then the leaf was immersed in a Petri dish containing distilled water for 24 h. After soaking, the surface moisture was removed using absorbent paper and the saturated weight (Wt) was recorded. Finally, the leaf was dried in an 80 °C oven for 48 h until a constant weight was achieved to obtain the dry weight (Wd). The relative water content (RWC) was then calculated using Formula (1).

RWC = (Wf − Wd)/(Wt − Wd) × 100%(1)

#### 2.3.2. Yield Determination

For each growth stage of *Cichorium intybus*, two 1 m × 1 m quadrats were randomly established within each plot. The stubble was maintained at a height of 3 cm. After mowing, the biomass was weighed to calculate the fresh herbage yield per unit area.

#### 2.3.3. Determination of Nutrient Elements

The nutrient element contents in the samples were determined using the methods described in the soil agrochemical analysis [22]. Calcium (Ca), phosphorus (P), magnesium (Mg), and potassium (K) were quantified by digesting the samples with nitric acid and perchloric acid, followed by an analysis with ICP or ICP-MS (inductively coupled plasma emission spectrometry).

#### 2.3.4. Determination of Nutritional Components

A 500 g sample of *Cichorium intybus* was collected from each of the nutritional, flowering, and seed-setting stages and brought back to the laboratory. The samples were first blanched in an oven at 105 °C for 30 min, and then dried at 65 °C until reaching a constant weight. Routine nutritional components were determined using the approximate nutrient analysis method [23] and the Van Soest detergent fiber analysis method [24]:

(1) The dry matter (DM) content was measured by drying in a thermostatic forced-air oven at 65 °C until a constant weight was achieved.
(2) Crude ash was determined by ashing the sample in a Nabertherm LE14/16/R6 (Nabertherm GmbH, Lilienthal, Germany) muffle furnace until reaching a constant weight.
(3) Crude fat (EE) was measured using an Ankom XT15 (ANKOM Technology Corporation, Macedon, NY, USA) fat-determination apparatus.
(4) Water-soluble carbohydrates (WSC) were measured using a colorimetric method with a UV spectrophotometer.
(5) Crude protein (CP) was analyzed using a FOSS Kjeltec 2300 (FOSS Analytical AB, Hillerød, Denmark) automatic protein analyzer.
(6) The neutral detergent fiber (NDF) and acid detergent fiber (ADF) were measured using the Van Soest method on an Ankom2000 fiber analyzer.

The results of the nutritional component determinations were used to evaluate the feeding value, including the total digestible nutrients (TDN), dry matter digestibility (DDM), dry matter intake (DMI), relative feeding value (RFV), and relative forage quality (RFQ). The calculations were performed following the formulas provided in [25].

TDN = 82.38 − (0.7515 × ADF)(2)

RFV = DMI × DDM ÷ 1.29(3)

RFQ = TDN × DMI ÷ 1.23(4)

DDM = 88.9 − (0.779 × ADF)(5)

DMI = 120 ÷ NDF(6)

### 2.4. Data Processing and Analysis

All data analyses were performed using SPSS 20.0 (IBM Corp., Armonk, NY, USA). A one-way ANOVA was employed to assess the effects of various forage indexes over different time periods, and inter-group differences were compared using the least significant difference (LSD) method (*p* < 0.05). Prior to hypothesis testing, the data were tested for normality and homogeneity of variances; if these assumptions were not met, non-parametric tests (such as the Kruskal–Wallis H test) were conducted as supplementary analyses. Graphs and charts were created using OriginPro 2023, and all results are presented as the mean ± standard error (Mean ± SE). The membership function method within fuzzy mathematics was used to comprehensively evaluate the morphological indexes, yield, and nutritional quality of *Cichorium intybus* over the three distinct periods, thereby obtaining the average membership value for *Cichorium intybus* at each period according to the following formula:

U(X) = (X − Xmin)/(Xmax − Xmin)(7)

U(X) = (Xmax − X)/(Xmax − Xmin)(8)

In the equation, X represents each feature value, Xmax denotes the maximum feature value, and Xmin denotes the minimum feature value. When the evaluation indicator is negative, the inverse membership function (Equation (8)) [26] should be employed.

## 3. Results

### 3.1. Analysis of Agronomic Traits of Cichorium intybus During Different Periods

#### 3.1.1. Analysis of Morphological Indexes of *Cichorium intybus* During Different Periods

**Figure 2:**
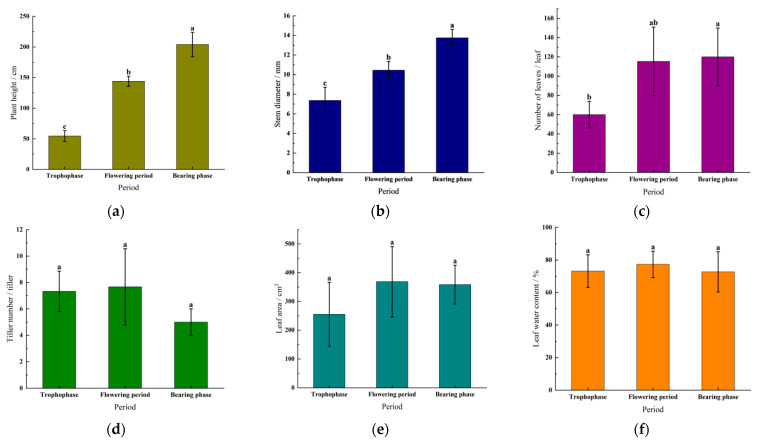
Morphological indexes of *Cichorium intybus* during different periods. Note: (**a**) is the plant height; (**b**) is the stem diameter; (**c**) is the number of leaves; (**d**) is the number of tillers; (**e**) is the leaf area; and (**f**) is the leaf moisture content. The lowercase letters indicate that there is a significant difference among the extracts of different mass concentrations (*p* < 0.05).

Agronomic traits are plant traits that directly affect crop yield, quality, and adaptability. They are important considerations in breeding and cultivation management. It can be seen from Figure 2a that the plant height of *Cichorium intybus* was significantly different during different growth stages, and the range of variation was 54.60–204.10 cm. The growth rate was the fastest during the transition from flowering to fruiting, and the net growth amount during this period was 60.10 cm. Based on Figure 2b, the stem diameter of *Cichorium intybus* also exhibited significant differences during different growth stages. The difference between the maximum stem diameter and the minimum stem diameter was 6.38 mm, and the stem diameter reached its peak during the fruiting stage, which was 13.75 mm. As shown in Figure 2c, the number of leaves of *Cichorium intybus* was between 60.00 and 120.00. Although the number of leaves fluctuated at different growth stages, there was no significant difference. It is worth noting that, from vegetative growth to flowering, the rate of increase in the leaf number was the highest, with a net increase of 55.33 leaves. Figure 2d reveals that the number of *Cichorium intybu* tillers during all growth stages varied, but did not differ significantly, with the highest number of tillers during the flowering stage at 7.67 and the lowest during the fruiting stage at 5.00. According to Figure 2e, although the difference in leaf area among the growth stages was not significant, the maximum leaf area occurred during the flowering stage at 368.57 cm^2^, and the minimum leaf area occurred during the nutrient stage at 255.16 cm^2^, representing a difference of 113.41 cm^2^. Finally, Figure 2f shows that the leaf moisture content varied among the growth stages, with the maximum value of 77.35% occurring during the flowering stage. The minimum value of 72.68% occurred during the fruiting stage, with a difference of 4.67%. *Cichorium intybu* grows fastest during the flowering and fruiting periods, especially during the fruiting period when the plant height and stem thickness are at their maximum. Flowering occurs when *Cichorium intybu* has the highest number of leaves and leaf area, and it is suitable for harvesting during this period, yielding a high number of leaves and a large leaf area. *Cichorium intybu* has the highest number of tillers during flowering, and this stage is an excellent time to manage for more tillers. The leaf moisture content was highest during flowering and lowest during fruiting, reflecting the changing water requirements of the plant at different growth stages.

#### 3.1.2. Analysis of *Cichorium intybus* Fresh Yield During Different Periods

**Figure 3:**
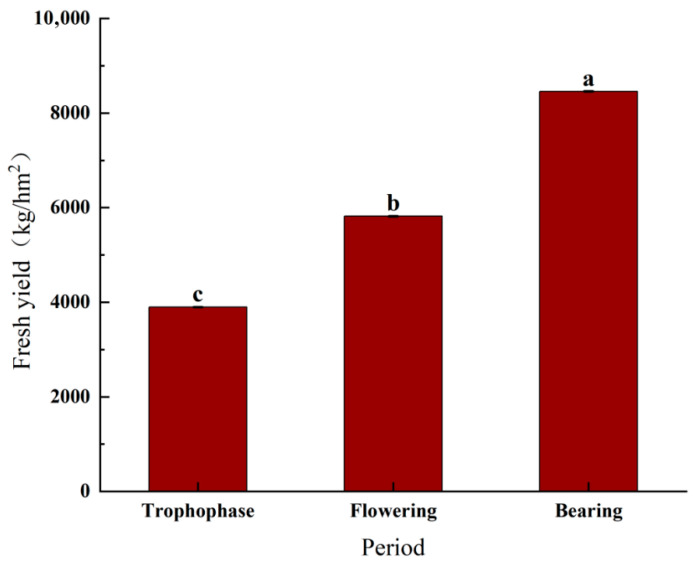
Fresh biomass yield of *Cichorium intybus* during different stages. Note: The lowercase letters indicate that there is a significant difference among the extracts of different mass concentrations (*p* < 0.05).

The fresh grass yield is the weight of fresh grass harvested from a given area of land over a given period and is often used to measure the productivity of a grass or forage crop. Figure 3 demonstrates the variation in the yield of *Cichorium intybu* during different growth stages. Significant differences (*p* < 0.05) were observed in the fresh grass yield during different growth stages. The fresh grass yield was significantly higher in the fruiting period than in the other two periods, while it was significantly lower in the nutritive period than in the other two periods. The lowest fresh grass yield of 8775.045 kg/hm^2^ was recorded during the nutrient stage. The fresh grass yield during the flowering stage increased significantly to 13,095.0675 kg/hm^2^, which was 4319.0225 kg/hm^2^ more compared to the nutrient stage. The highest fresh grass yield was reached during the fruiting stage, with 19,035.09 kg/hm^2^, which increased by 5940.0225 kg/hm^2^ compared to the yield during the flowering stage and 10,260.045 kg/hm^2^ compared to that during the nutrient stage. From the point of view of fresh grass yield, the fruiting stage is the best time for harvesting, as it results in the highest fresh grass yield and provides more feed resources.

### 3.2. Analysis of Nutritional Quality of Cichorium intybus Grass in Different Periods

#### 3.2.1. Analysis of Nutritional Indexes of *Cichorium intybus* Grass in Different Periods

Nutritional indicators are important parameters used to assess the nutritional status of plants. Based on the data analyzed in Table 1, the nutritional indicators of *Cichorium intybus* grass stems and leaves during different growth stages showed significant differences. The dry matter (DM) content of the stems and leaves showed a gradual increase as the growth cycle of the plant advanced. Specifically, the stem dry matter (DM) content increased from a minimum of 8.73% to a maximum of 20.52% during the fruiting stage, exhibiting an increase of 11.79% compared to the nutrient stage. In comparison, the leaf dry matter (DM) content increased from 14.04% to 20.31% at the fruiting stage, representing an increase of 6.27%.

Meanwhile, the stem and leaf crude ash (Ash) content decreased gradually over time. The crude ash (Ash) content in the stems ranged from 7.86 to 23.30% and was the highest during the nutrient stage—significantly higher than the other periods at 23.30%. The crude ash (Ash) content of the leaves ranged from 16.56 to 19.49% and was the highest during the nutritive period, at 19.45%. The crude fat (EE) content of the stems decreased over time from 2.95% to a minimum of 1.95% during the nutritive stage; the crude fat (EE) content of the leaves reached a peak of 5.02% during the flowering stage, which was 2.33% higher than the crude fat (EE) content of the leaves during the nutritive stage. In addition, the crude protein (CP) content decreased over time, with a 7.32% reduction in the stems from 12.02% during the nutrient stage to a minimum value of 4.70% during the fruiting stage. The leaf crude protein (CP) content decreased by 9.35%, from 20.32% during the nutrient stage to the lowest value of 10.97% during the fruiting stage.

The acid detergent fiber (ADF) and neutral detergent fiber (NDF) contents in the stems increased over time. The stem’s acidic detergent fiber (ADF) content reached a maximum value of 42.43% during the fruiting stage, which represented an increase of 8.2% compared to that during the nutrient stage. The neutral detergent fiber (NDF) content reached a maximum value of 53.13% during the fruiting stage, which represented an increase of 12.08% compared to that during the nutrient stage. The acid detergent fiber (ADF) and neutral detergent fiber (NDF) contents of the leaves likewise reached maximum values of 24.45% and 31.03%, respectively, during the fruiting stage, representing increases of 3.77% and 4.54%, respectively, compared to the values during the nutrient stage. Finally, the stem’s soluble carbohydrate (WSC) content decreased with time, reaching a minimum value of 13.13% during the fruiting stage, which was 7.92% less compared to that during the nutrient stage. In contrast, the leaf soluble carbohydrate (WSC) content reached a maximum value of 7.65% during the fruiting stage, which was 3.95% higher than that during the flowering stage.

#### 3.2.2. Comparison of Nutrient Elements During Different Growth Periods of *Cichorium intybus* Grass

Nutrients are essential for proper plant growth and development. As shown in Table 2, the nutrient elements in *Cichorium intybus* grass stems and leaves exhibited different performances at different times. The maximum calcium (Ca) content in both the stems and leaves occurred during the nutrient stage, with values of 2.04% and 2.61%, respectively. The minimum values occurred during the flowering stage, at 1.36% and 2.24%, respectively, with a difference of 0.68% and 0.37% between the maximum and minimum values. The phosphorus (P) content in the stems showed a decreasing trend with time, with the maximum value occurring during the nutrient stage at 0.44% and the minimum value occurring during the fruiting stage at 0.14%, with a difference of 0.30%. The maximum phosphorus (P) content of leaves was observed during the nutrient stage at 0.46%, and the minimum value occurred during the flowering stage at 0.29%, with a difference of 0.17%. The magnesium (Mg) content in the stems showed a decreasing trend with time; the maximum value appeared during the nutrient stage at 0.69% and the minimum value appeared during the fruiting stage at 0.33%, with a difference of 0.36%. The magnesium (Mg) content in the leaves showed an increasing trend with time, with the maximum value occurring during the fruiting stage at 0.57% and the minimum during the nutrient stage at 0.43%, with a difference of 0.14%. The potassium (K) content in the stems showed a decreasing trend with time; the maximum value appeared during the nutrient stage, with 3.62%, and the minimum value appeared during the fruiting stage, with a difference of 1.76%. The maximum potassium (K) content in the leaves appeared during the flowering stage at 4.66% and the minimum value during the fruiting stage at 4.49%, with a difference of 0.17%.

#### 3.2.3. Analysis of Relative Feeding Value and Relative Forage Quality of *Cichorium intybus* Grass During Different Periods

The relative feeding value and relative forage quality are essential forage-intake and energy-value measures. As shown in Table 3, the maximum values of the total digestible nutrients (TDN), dry matter digestibility (DDM), dry matter intake (DMI), relative feeding value (RFV), and relative forage quality (RFQ) of the stems and leaves occurred during the nutrient period. All the leaf indexes were more significant than those of the stems. As the growth cycle increased, all indicators showed a downward trend. The total digestible nutrients (TDN) in the stem decreased by 6.17%, from 56.60% during the nutrient stage to 50.43% during the fruiting stage, while the total digestible nutrients (TDN) in the leaf decreased by 2.08%, from 66.78% during the nutrient stage to 64.70% during the fruiting stage. The dry matter digestibility (DDM) of the stems decreased by 6.38%, from 62.23% during the nutrient stage to 55.85% during the fruiting stage, while that of the leaves decreased by 2.16%, from 72.79% during the nutrient stage to 70.63% during the fruiting stage. The dry matter intake (DMI) of the stems decreased by 0.66 kg/day, from 2.92 kg/day in the trophic stage to 2.26 kg/day in the fruiting stage, and that of leaves decreased by 0.66 kg/day, from 4.53 kg/day in the trophic stage to 3.87 kg/day in the fruiting stage. The relative feeding value (RFV) of the stems declined by 43.45, from 141.03 during the trophic stage to 97.78 during the fruiting stage, while that of leaves declined by 43.68, from 255.61 during the trophic stage to 211.75 during the fruiting stage. The relative forage quality (RFQ) of the stems decreased by 41.90, from 134.51 during the nutrient stage to 92.61 during the fruiting stage, while the relative forage quality (RFQ) of the leaves decreased by 42.53, from 245.94 during the nutrient stage to 203.41 during the fruiting stage. The relative feeding value (RFV) and relative forage quality (RFQ) of *Cichorium intybus* grasses during different growth stages reached a maximum during the nutrient stage, followed by a gradual decline. The total digestible nutrients (TDN), dry matter digestibility (DDM), and dry matter intake (DMI) were also the highest during the nutrient period. These results suggest that the nutrient period is the optimal harvest time for *Cichorium intybus* grasses, with harvests during this period yielding the highest feeding value and forage quality.

### 3.3. Correlation Analysis

#### 3.3.1. Correlation Analysis of *Cichorium intybus* Agronomic Traits During Different Periods

In order to explore the correlation among the agronomic traits of *Cichorium intybus* during different growth stages, this study carried out a correlation analysis on agronomic traits such as the plant height, stem diameter, leaf number, tiller number, leaf area, leaf relative water content, and fresh grass yield.

**Figure 4:**
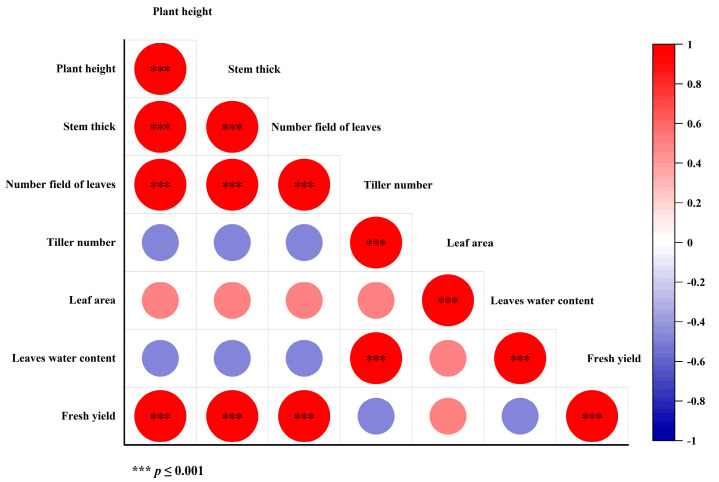
Correlation among agronomic traits of *Cichorium intybus* during different periods.

As shown in Figure 4, there was a significant positive correlation between the plant height and the stem diameter, leaf number, and fresh grass yield (*p* < 0.001). The corresponding R^2^ was 0.89, 0.82, and 0.91, respectively, indicating that plant height was highly correlated with these three traits. The linear fitting degree with the fresh grass yield was the highest. The stem diameter was also significantly positively correlated with the leaf number and fresh grass yield (*p* < 0.001), and R^2^ was 0.78 and 0.85, respectively, indicating that the stem diameter exhibited a high degree of explanation for the leaf number and fresh grass yield. At the same time, the number of leaves and the fresh grass yield also showed a very significant positive correlation (*p* < 0.001); the R^2^ was 0.75, reflecting a strong synergistic trend between the two. In addition, the R^2^ values for the tiller number, leaf area, leaf relative water content, and other agronomic traits were lower than 0.5, and the correlation degree was relatively weak.

#### 3.3.2. Correlation Analysis Between Morphological Indexes and Nutritional Quality of *Cichorium intybus* Grass During Different Periods

In order to clarify the correlation between agronomic traits and the nutritional quality of *Cichorium intybus* during different growth stages, this study selected morphological indexes such as the plant height, stem diameter, tiller number, leaf number, leaf area, leaf water content, and fresh grass yield. A correlation analysis was performed on nutritional indicators such as the stem and leaf dry matter (DM), crude ash (Ash), crude fat (EE), crude protein (CP), acid detergent fiber (ADF), neutral detergent fiber (NDF), soluble carbohydrate (WSC), calcium (Ca), phosphorus (P), magnesium (Mg), potassium (K), total digestible nutrients (TDN), dry matter digestibility (DDM), dry matter intake (DMI), relative feed value (RFV), and relative feed quality (RFQ).

**Figure 5:**
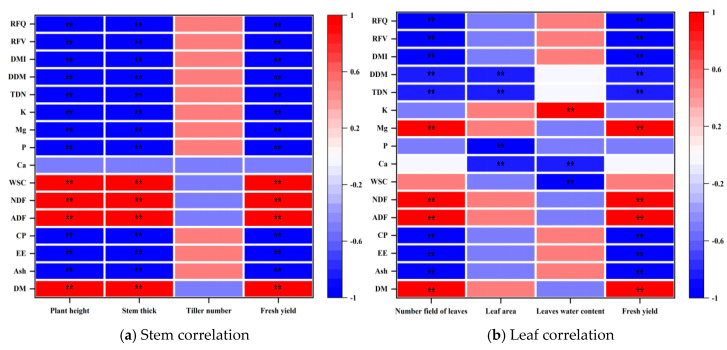
Correlation between agronomic traits and nutritional quality of *Cichorium intybus* during different periods. ** Significant correlation at 0.01 (two-tailed).

It can be seen from Figure 5a that the plant height, stem diameter, and fresh grass yield were significantly positively correlated with the dry matter content, acid detergent fiber, neutral detergent fiber, and soluble carbohydrates (*p* < 0.01), which indicated that the changes in the morphological indexes were synchronously correlated with the accumulation of these nutrients. However, it does not mean that the development of morphological indexes directly led to these changes in nutrients.

It can be seen from Figure 5b that the number of leaves was significantly positively correlated with the dry matter content, acid detergent fiber, neutral detergent fiber, and magnesium content (*p* < 0.01). The leaf water content was significantly positively correlated with the potassium content (*p* < 0.05), and the fresh grass yield was also significantly positively correlated with the magnesium content (*p* < 0.05). These results only prove that there is a correlation trend between related indicators. For example, when the number of leaves increases, the magnesium content often increases, but it cannot be confirmed that the increase in the number of leaves was a direct cause of the increase in the magnesium content. The internal correlation may be driven by common growth regulators or environmental conditions, rather than direct causal effects.

Overall, the plant height, stem thickness and leaf number were more closely related to some nutritional indexes, which could be used as important reference indexes to reflect changes in the nutritional quality of *Cichorium intybus*. However, it should be clarified that this correlation is only the corresponding relationship at the data level, rather than the causal relationship at the physiological level.

### 3.4. Membership Function Analysis

In order to comprehensively assess the overall performance of *Cichorium intybus* grass during the three different growth periods, a series of indicators, including the plant height, stem thickness, number of tillers, fresh herbage yield, dry matter, crude ash, crude fat, crude protein, acid detergent fiber, neutral detergent fiber, soluble carbohydrates, calcium, phosphorus, magnesium, and potassium, were used to comprehensively evaluate the stems of *Cichorium intybus* grass using an affiliation function in this study. According to the data in Table 4, stems in the nutrient stage performed the best, with an affiliation function value of 0.54, followed by stems in the flowering stage, with an affiliation function value of 0.50, and finally, stems in the fruiting stage exhibited the worst performance, with an affiliation function value of 0.49. Similarly, in order to develop a more accurate picture of the performance of *Cichorium intybus* grass leaves during different growth periods, we used the number of leaves, leaf area, leaf water content, fresh herbage yield, dry matter, crude ash, crude fat, crude protein, acid detergent fiber, neutral detergent fiber, soluble carbohydrates, calcium, phosphorus, magnesium, and potassium in a comprehensive evaluation of the foliar parts of the *Cichorium intybus* grass as a result of the affiliation function. According to the results of Table 5, the leaves in the vegetative period showed the best performance, and the membership function value was 0.60. This was followed by the leaves in the flowering stage, with a membership function value of 0.52, and finally by the leaves in the fruiting period, with a membership function value of 0.50.

## 4. Discussions

This study conducted a systematic analysis of the agronomic traits, the nutritional quality, and their interrelationships across three critical growth stages—vegetative, flowering, and fruiting—of *Cichorium intybus*, thereby revealing the dynamic variations in this plant’s growth patterns and fodder value. The following discussion delves into these aspects from three distinct perspectives.

### 4.1. Differential Analysis of Cichorium intybus During Different Growth Stages

*Cichorium intybus* exhibits significant differentiation in its morphological characteristics, fresh forage yield, nutritional value, and production efficiency across its various growth stages, which directly reflects its adaptation to growth demands and resource allocation strategies. In terms of morphological traits, indicators such as the plant height and stem diameter show a consistent increase throughout the growth cycle: the plant height during the vegetative stage is only 54.60 cm, but increases to 204.10 cm during the seed-setting stage, with a net increment of 60.10 cm from the flowering stage to the seed-setting stage. This increase is associated with the need for stem elongation to provide space and support for seed development once the plant enters the reproductive phase [27,28]. Similarly, the stem diameter reaches 13.75 cm at the seed-setting stage, a 6.38 cm increase compared to the vegetative stage, reflecting a marked rise in the demand for mechanical strength and nutrient transport capacity during reproduction. Although there were no significant differences in the number of leaves and tillers, a net increase of 55.33 leaves and 7.67 tillers during the flowering stage suggests that this is a critical period for the expansion of the photosynthetic area, thereby laying the foundation for subsequent biomass accumulation. Furthermore, the maximum leaf area during the flowering stage reached 368.57 cm^2^, confirming that the plant’s demand for light energy capture peaks at this time [29].

In terms of fresh forage yield, the seed-setting stage achieved the highest yield (19,035.09 kg/hm^2^), which was a more than 117% increase compared to the yield during the vegetative stage (8775.05 kg/hm^2^). This yield enhancement is directly related to the full development of the plant’s morphology—improvements in plant height, stem diameter, and leaf area collectively create a larger space for biomass accumulation and a stronger photosynthetic capacity. However, it should be noted that the yield and nutritional quality do not increase synchronously; for instance, although the yield during the vegetative stage is the lowest, its nutritional composition is superior, reflecting the plant’s trade-off strategy between “growth” and “nutrition” allocation [30].

Regarding the nutritional value, the core differences across the three stages lie in the functional orientation of the nutritional components. During the vegetative stage, the dry matter (DM) content is low (stems: 8.37%; leaves: 14.04%), yet the crude protein (CP) and ether extract (EE) contents are the highest (leaf: CP, 20.32%; EE, 5.02%). This is because the plant is dominated by vegetative growth during this period, requiring large amounts of protein synthesis for cell division and tissue differentiation, while fats serve as energy reserves to support rapid growth. During the flowering stage, the content of water-soluble carbohydrates (WSC) significantly increases (reaching 7.65% in the leaves), which is related to the surge in energy consumption during flowering—carbohydrates act as a rapid energy source to meet the demands of reproductive organ development. In the seed-setting stage, the dry matter (stems: 20.52%; leaves: 20.31%), acid detergent fiber (ADF), and neutral detergent fiber (NDF) contents are the highest (stem: ADF, 42.43%; NDF, 53.13%), while the crude protein content is the lowest (leaf: 10.97%). This indicates that, at this stage, the plant’s resource allocation shifts toward seed maturation; the increase in fibrous substances enhances the supportive capacity of the stems, whereas the reduction in protein synthesis is due to more energy being devoted to the accumulation of structural materials (such as cellulose) in the seeds [31].

In terms of the production efficiency, the vegetative stage exhibits the highest relative feeding value (RFV) and relative forage quality (RFQ) for leaves (RFV: 255.61; RFQ: 245.94), indicating the most efficient utilization of biomass for feed purposes. Although the seed-setting stage yields the highest overall production, both the RFV and RFQ decline by over 43%, suggesting that the high yield is accompanied by a certain loss in quality, and the production efficiency must therefore be evaluated comprehensively in relation to the product’s intended use [32].

### 4.2. Mechanism Underlying the Correlation Between Agronomic Traits and Nutritional Quality

The correlation analysis revealed an intrinsic connection between the growth and development of *Cichorium intybus* and nutrient accumulation, reflecting both the synergistic and restrictive aspects of plant physiological metabolism. Among the agronomic traits, the plant height, stem diameter, and leaf number exhibited a highly significant positive correlation with fresh herbage yield (*p* < 0.001), with the correlation coefficients exceeding 0.8. This indicates that the coordinated enhancement of morphological indicators is a key driving factor for yield improvement—an increase in plant height expands the growth space, a thicker stem enhances the nutrient transport capacity, and a greater number of leaves boosts the photosynthetic efficiency, all contributing jointly to biomass accumulation [33]. The correlation between the tiller number and the yield was weak (*p* > 0.05), suggesting that yield formation in *Cichorium intybus* relies more on the growth of the main stem rather than on the expansion of tillers, a characteristic associated with its perennial herbaceous nature [34]. In the analysis of correlations between morphological indicators and the nutritional quality, the plant height and stem diameter showed significant positive correlations with the dry matter (DM), acid detergent fiber (ADF), and neutral detergent fiber (NDF) (*p* < 0.01), indicating that, as the plant grows larger, there is a concomitant accumulation of fibrous substances. This is because the degree of lignification in the stem intensifies over the growth period, leading to an increased proportion of cell wall components (such as cellulose and hemicellulose) [35]. The positive correlation between fresh herbage yield and the DM, ADF, and NDF (*p* < 0.01) further confirms that higher yields are often accompanied by an increase in structural substances, which also explains why high yields during the seed-setting stage are associated with a reduced digestibility [36]. Moreover, the number of leaves was positively correlated with the magnesium (Mg) content (*p* < 0.05), as magnesium is a core component of chlorophyll and an increased number of leaves increases the magnesium requirement for the synthesis of photosynthetic pigments [37]. The water content in the leaves was positively correlated with the potassium (K) content (*p* < 0.05), which was closely linked to the key role of potassium in regulating the plant water status (maintenance of cellular osmotic pressure).

### 4.3. Determination of the Optimal Harvesting Period

Based on a comprehensive evaluation using membership functions (with the membership values for stems and leaves during the nutritional phase being 0.54 and 0.60, respectively, and both the highest) and the characteristics of each period, the applicable scenarios for *Cichorium intybus* vary distinctly during different growth stages; consequently, the selection of the optimal harvest time should be tailored to the feeding objectives and resource requirements.

The primary advantage of the nutritional phase lies in its high nutrient density: the crude protein and crude fat contents are significantly higher than in other stages, while the fiber content is low (ADF in the leaves at 20.68% and NDF at 26.49%), and the dry matter digestibility (DDM) reaches 72.79%. This stage is therefore suitable for feeding scenarios that urgently require high-protein, high-digestibility feed, such as for young livestock or high-yielding dairy cows. Although the yield at this stage is relatively low, harvesting at this time maximizes the expression of the plant’s “quality” characteristics [38].

The flowering stage is characterized by a remarkable nutritional balance: the dry matter content is moderate (16.80% in stems and 19.12% in leaves), and there is a significant accumulation of water-soluble carbohydrates (WSC). When harvested during this stage, the plant can be combined with low-energy feeds (such as straw) so that the supplementary carbohydrates enhance both the palatability and the energy level of the mixed feed, making it appropriate as transitional feed for fattening livestock [39].

During the fruiting stage, the high dry matter and fiber characteristics render the material more suitable for processing and storage: the dry matter content exceeds 20%, and both the acid detergent fiber (ADF) and neutral detergent fiber (NDF) contents are high. Following ensiling or sun-drying, it is less prone to mold, and the fiber promotes rumen motility in ruminants, making it suitable as winter reserve feed or as a basal feed for adult livestock.

In summary, the next research direction is as follows: Multi-regional and multi-year field verification tests can be carried out for different *Cichorium intybus* varieties to clarify the interaction effect between varieties and the regional environment, and to screen special forage *Cichorium intybus* varieties suitable for cold and arid regions in the north.

The synthetic metabolic pathways of key nutrients (such as functional active substances) in different growth stages of *Cichorium intybus* should be deeply explored, and the molecular regulation mechanism of a “yield–quality” synergistic improvement should be analyzed to provide theoretical support for directional breeding.

A livestock feeding experiment using *Cichorium intybus* forage harvested at different stages could also be carried out to quantify the actual effects of *Cichorium intybus* forage at different growth stages on the livestock production performance and intestinal health, and to construct a full-chain technical system of “*Cichorium intybus* cultivation–feed conversion–livestock fattening”.

## 5. Conclusions

This study clarified the dynamic differences and internal relationships between the agronomic traits and nutritional quality of *Cichorium intybus* during the three key stages—the vegetative growth period, flowering period, and fruiting period—in a northern temperate continental monsoon climate zone (Hohhot). The comprehensive performance of the stems and leaves of *Cichorium intybus* during the vegetative growth period was the best. The contents of crude protein (20.32%) and crude fat (5.02%) in the leaves were the highest, and the fiber content was the lowest. The dry matter digestibility (72.79%) and relative feed value (255.61) were at their peak; thus, this was the best harvesting period for producing high-quality forage with a high protein content and high digestibility. The fresh yield of *Cichorium intybus* in the flowering stage (13,095.07 kg/hm^2^) was significantly higher than that during the vegetative growth stage, and the water-soluble carbohydrate content was moderate. When harvested at this time, the plant could be used as a transitional mixed feed raw material for livestock during a fattening period. The fresh grass yield of *Cichorium intybus* reached its peak (19,035.09 kg/hm^2^), and the dry matter and fiber content were the highest. Although the crude protein content was the lowest, it was suitable for processing as hay or silage for winter forage reserve. At the same time, the correlation analysis confirmed that the plant height, stem diameter, and leaf number were the core agronomic traits driving the increase in the *Cichorium intybus* yield, and the development of agronomic traits was significantly positively correlated with the accumulation of fiber substances, revealing the physiological regulation mechanism of the *Cichorium intybus* “yield–quality” trade-off.

## Figures and Tables

**Table 1:** Nutritional indexes of *Cichorium intybus* grass during different periods.

Item		DM/%	Ash/%	EE/%	CP/%	ADF/%	NDF/%	WSC/%
Stem	Trophophase	8.73 ± 0.50 c	23.30 ± 0.64 a	2.95 ± 0.56 a	12.02 ± 0.84 a	34.23 ± 0.75 c	41.05 ± 1.86 c	5.21 ± 0.51 c
	Flowering period	16.80 ± 0.62 b	9.46 ± 0.57 b	2.39 ± 0.77 a	5.53 ± 0.64 b	40.50 ± 0.70 b	49.49 ± 0.78 b	11.67 ± 0.43 b
	Bearing phase	20.52 ± 0.69 a	7.86 ± 1.02 c	1.95 ± 0.82 a	4.70 ± 0.76 b	42.43 ± 1.01 a	53.13 ± 1.02 a	13.13 ± 0.39 a
Leaf	Trophophase	14.04 ± 0.62 B	19.45 ± 0.65 A	5.02 ± 0.86 A	20.32 ± 0.65 A	20.68 ± 1.09 A	26.49 ± 0.93 B	5.77 ± 0.33 B
	Flowering period	19.12 ± 0.64 A	18.26 ± 0.93 A	4.18 ± 0.35 B	13.20 ± 0.41 B	23.45 ± 0.65 A	30.72 ± 0.45 A	3.70 ± 0.34 C
	Bearing phase	20.31 ± 1.14 A	16.56 ± 0.72 B	2.69 ± 0.53 A	10.97 ± 0.65 C	24.45 ± 3.18 A	31.03 ± 2.98 A	7.65 ± 0.67 A

Note: Different uppercase/lowercase letters indicate that there is a significant difference among the extracts of different mass concentrations (*p* < 0.05).

**Table 2:** Nutrient elements of *Cichorium intybus* grass during different periods.

Item		Ca/%	P/%	Mg/%	K/%
Stem	Trophophase	2.04 ± 0.11 a	0.44 ± 0.07 a	0.69 ± 0.09 a	3.62 ± 0.33 a
	Flowering period	1.36 ± 0.06 b	0.21 ± 0.02 b	0.37 ± 0.03 b	2.07 ± 0.06 b
	Bearing phase	1.51 ± 0.17 b	0.14 ± 0.03 b	0.33 ± 0.04 b	1.86 ± 0.06 b
Leaf	Trophophase	2.61 ± 0.06 A	0.46 ± 0.03 A	0.43 ± 0.04 B	4.52 ± 0.05 B
	Flowering period	2.24 ± 0.04 B	0.29 ± 0.05 B	0.53 ± 0.07 AB	4.66 ± 0.06 A
	Bearing phase	2.61 ± 0.03 A	0.35 ± 0.03 B	0.57 ± 0.06 A	4.49 ± 0.08 A

Note: Different uppercase/lowercase letters indicate that there is a significant difference among the extracts of different mass concentrations (*p* < 0.05).

**Table 3:** Nutritional value of *Cichorium intybus* grass during different periods.

Item		TDN/%	DDM/%	DMI (kg/day)	RFV	RFQ
Stem	Trophophase	56.60	62.23	2.92	141.03	134.51
	Flowering period	51.88	57.35	2.42	107.80	102.28
	Bearing phase	50.43	55.85	2.26	97.78	92.61
Leaf	Trophophase	66.78	72.79	4.53	255.61	245.94
	Flowering period	64.70	70.63	3.91	213.88	205.47
	Bearing phase	64.70	70.63	3.87	211.75	203.41

**Table 4:** Evaluation of agronomic traits and nutritional quality membership function of *Cichorium intybus* stems during different periods.

Item	Plant Height	Stem Thickness	Tiller Number	Fresh Yield	DM	Ash	EE	CP	ADF	NDF	WSC	Ca	P	Mg	K	Mean Value
Trophophase	0.61	0.41	0.67	0.69	0.52	0.56	0.49	0.76	0.51	0.66	0.44	0.38	0.42	0.46	0.60	0.54
Flowering period	0.41	0.35	0.33	0.43	0.48	0.51	0.63	0.60	0.50	0.45	0.52	0.61	0.50	0.57	0.59	0.50
Bearing phase	0.61	0.32	0.67	0.35	0.64	0.44	0.41	0.53	0.53	0.43	0.56	0.60	0.38	0.38	0.56	0.49

**Table 5:** Evaluation of membership function of agronomic traits and nutritional quality of *Cichorium intybus* leaves during different periods.

Item	Number of Leaves	Leaf Area	Leaf Water Content	Fresh Yield	DM	Ash	EE	CP	ADF	NDF	WSC	Ca	P	Mg	K	Mean Value
Trophophase	0.56	0.44	0.46	0.69	0.48	0.63	0.57	0.48	0.57	0.54	0.78	0.86	0.83	0.38	0.80	0.60
Flowering period	0.45	0.39	0.41	0.43	0.50	0.63	0.43	0.49	0.56	0.57	0.59	0.52	0.83	0.60	0.42	0.52
Bearing phase	0.50	0.44	0.58	0.50	0.43	0.65	0.54	0.39	0.38	0.40	0.37	0.50	0.57	0.46	0.85	0.50

## Data Availability

The original contributions presented in this study are included in the article; further inquiries can be directed to the corresponding author.
